# Leveraging artificial intelligence to enhance marine biosecurity

**DOI:** 10.1093/biosci/biag049

**Published:** 2026-05-01

**Authors:** Marnie L Campbell, Chi T U Le, Jumana Abu-Khalaf, Craig D H Sherman

**Affiliations:** School of Science, Edith Cowan University, 270 Joondalup Drive, Joondalup, WA 6027, Australia; School of Science, Edith Cowan University, 270 Joondalup Drive, Joondalup, WA 6027, Australia; School of Science, Edith Cowan University, 270 Joondalup Drive, Joondalup, WA 6027, Australia; School of Life and Environmental Science, Deakin University, 75 Pigdons Rd, Waurn Ponds, VIC 3216, Australia

**Keywords:** innovation in marine pest management, non-native marine species, inclusive and equitable AI, global collaboration, marine ecosystem protection

## Abstract

Marine biosecurity stands at the crossroads of innovation and necessity, with artificial intelligence (AI) offering promising tools to enhance risk assessment, surveillance, and response strategies for invasive species management. Despite the rapid growth of AI applications in marine science, there has been no comprehensive overview of its potential role in enhancing and supporting marine biosecurity. Our literature review aims to fill this gap, showing that AI use in this field remains limited and underdeveloped. Through our literature analysis, we identify key opportunities for AI research and innovation across the marine biosecurity continuum, while also highlighting both general and domain-specific challenges. We conclude by proposing a roadmap to guide the responsible development and implementation of AI in marine biosecurity.

## Glossary

**Marine biosecurity**: A suite of management actions taken by a jurisdiction to prevent and minimize the introduction, establishment, and spread of non-indigenous marine species (NIMS).

**Pathway:** The processes/mechanisms through which non-indigenous species are transferred to a novel environment (Hulme et al. 2008).

**Profiling:** The analysis to identify if an entity is “risky” based on prior information, experience, or beliefs to prioritize intervention activities (Mannix et al. 2024).

**Risk:** The product of likelihood and consequence of an event, derived by multiplying likelihood by consequence (risk = likelihood x consequence) (Hester et al. 2024).

**Risk assessment:** Estimation of risks, either qualitative or quantitative. Qualitative risk assessment is based on subjective information and judgements, which in turn are based on experience, evidence, and expert opinions. In quantitative risk assessment, numerical estimates of risk are based on numerical values derived from data or expert judgement, often using mathematical formulae or quantitative models (Hester et al. 2024).

**Vector:** The physical means of transport by which non-indigenous species are transferred from one location to another (Hulme et al. 2008).

*NB:* There is a lack of consistent use of the terms “vector” and “pathway” in the bioinvasion field, including marine bioinvasions. In some cases, the terms “vector” and “pathway” are used interchangeably to describe the same phenomenon, depending on the context. For example, “shipping” and “hull fouling” have been referred to as both pathways and vectors in various studies and reports (Gollasch 2002, Godwin 2003, Seebens et al. 2013, Williams et al. 2013, CBD 2014, Ojaveer et al. 2017, Laeseke et al. 2020, Jensen et al. 2023). The definitions of the terms adopted in this paper are for simplicity, rather than representing standardized terminology.

## Introduction

Marine bioinvasions occur in vast and highly connected environments, making them transboundary in nature, with complex interconnections and far-reaching ecological, economic, and societal impacts (Alidoost Salimi et al. 2021, Carvalho et al. 2023, Campbell et al. 2024). NIMS can be transported outside their home ranges through both natural processes, such as ocean currents, storms, and tsunami, and human-mediated activities, whether intentional (e.g., importing for aquaculture purposes), or unintentional (e.g., ballast water discharge) (Rilov and Crooks 2009, Campbell 2011, Ojaveer et al. 2017). Among human-mediated pathways, global maritime activities, including shipping, trade, and tourism, play a significant role, with ballast water and hull fouling acting as primary vectors for species transfer across coastal and marine ecosystems worldwide (Seebens et al. 2013, Ros et al. 2023). The success of the introduction, establishment, and spread of NIMS depends on various environmental, ecological, and anthropogenic factors, making marine bioinvasions dynamic, highly complex, and context-dependent (Papacostas et al. 2017).

Despite a limited understanding of how NIMS interacts with local biotic and abiotic environments and their long-term consequences (Bonanno and Orlando-Bonaca 2019), mounting evidence highlights their detrimental impacts on biodiversity, habitat structure, human well-being, and socio-economic stability (Alidoost Salimi et al. 2021, Tsirintanis et al. 2022, Tsirintanis et al. 2023, Campbell et al. 2024). Globally, these impacts are expected to intensify due to climate change and accelerating anthropogenic pressures (Essl et al. 2020, Iacarella et al. 2020, Hulme 2021, Occhipinti-Ambrogi 2021). Adding to the challenge, the difficulty of underwater monitoring means that marine bioinvasions often go undetected until they are established and their populations are well-advanced, making them nearly impossible to contain (Beric and MacIsaac 2015). Therefore, effective risk assessment and early detection are crucial in protecting marine integrity and ecosystem services.

There is an urgent need for practical, cost-effective marine biosecurity programs that require proactive and quick actions, emphasizing prevention and early detection with predictive capabilities to account for climate and anthropogenic changes (Cook et al. 2016, Hayes et al. 2019, Collin and Shucksmith 2022). Since control and eradication are extremely difficult in marine environments, proactive approaches to prevent the arrival and establishment are the most effective means of reducing marine bioinvasion impacts (Locke and Hanson 2009). This calls for innovation beyond traditional methods to improve risk assessments, detection, and management of NIMS.

Artificial intelligence (AI) is rapidly emerging as a transformative tool in marine science, management, and conservation, with significant potential to advance the understanding and protection of ocean ecosystems (Beyan and Browman 2020, Ditria et al. 2022, Fava et al. 2023, Song et al. 2023, Alloghani 2024, Chau et al. 2024). Defined as computational techniques that enable machines to process large datasets, recognize patterns, generate insights, and support decision-making with minimal human input (Wirtz et al. 2019), AI has facilitated notable advances across the marine sciences. Applications include autonomous underwater robots for non-intrusive exploration and automated data collection (Wang et al. 2021, Yoerger et al. 2021), reconstruction of incomplete or erroneous datasets (Ducournau and Fablet 2016, Durán-Rosal et al. 2016, Park et al. 2019, Li et al. 2021), enhanced sensor networks (Howe et al. 2010, Luo et al. 2022), and improved remote sensing data processing (Ball et al. 2017, Boukabara et al. 2019). AI also strengthens big data analysis, reducing bias and supporting evidence-based decisions (Ditria et al. 2022, Song et al. 2023). In marine pollution management, it improves monitoring anomaly detection, prediction, and response with greater precision and efficiency (Ning et al. 2024). Collectively, these advancements increase autonomy and accuracy, enabling more effective stewardship of our ocean resources (Ditria et al. 2022, Song et al. 2023). As AI continues to evolve, its integration into marine science and management holds immense promise for addressing these and other complex challenges, optimizing resource allocation, and safeguarding marine ecosystems.

Given AI’s expanding role in marine science, its application to biosecurity presents substantial opportunities. AI-driven automation, advanced data processing, and predictive modeling could enhance surveillance, risk assessment, and response strategies for invasive species management (Shafiq et al. 2024). Yet, despite these prospects, no comprehensive synthesis of AI applications in marine biosecurity currently exists. Addressing this gap is essential to evaluate progress, identify limitations, and guide future research and implementation in safeguarding marine ecosystems.

Applying AI in marine biosecurity requires careful consideration of domain-specific challenges (Otts 2015, Coughlan et al. 2020, Carvalho et al. 2023). Key constraints include difficulties in prevention and early detection, knowledge and data gaps regarding biological introductions, and limited resources for monitoring and management (Cook et al. 2016, Collin and Shucksmith 2022, Carvalho et al. 2023). Inconsistent regulations, enforcement mechanisms, risk assessment criteria, and stakeholder engagement across regions further impede coordinated action against NIMS (Hayes et al. 2019, Carvalho et al. 2023). Limited baseline biodiversity data, shortages of taxonomic expertise, and uncertainties surrounding pathways, vectors, and ecological traits of NIMS all hinder effective definitions, classification, and prediction (Carvalho et al. 2023). These challenges are compounded by insufficient investment from governments and industries (Scott and McGill 2019, Johansen and Vestvik 2020, Andriamahefazafy et al. 2022). Nonetheless, they also highlight areas for research innovation (Carvalho et al. 2023).

Building on established applications of AI in the marine domain, this review seeks to examine how AI can strengthen biosecurity practices, considering these challenges. We assess the current state of AI applications in marine biosecurity, identify opportunities for integration, and discuss ethical and practical barriers to implementation. Finally, we propose a roadmap for advancing AI in marine biosecurity to maximize the benefits of technological innovation.

## A literature review

Marine biosecurity stands at the crossroads of innovation and necessity, demanding strategic integration of AI to bolster its effectiveness. An overview of marine biosecurity is presented in box 1 with a marine biosecurity continuum that serves as a conceptual framework for the subsequent literature review.

Box 1. A brief introduction to the marine biosecurity context.

As part of a holistic biosecurity system, marine biosecurity represents management efforts in marine, estuarine, coastal, and oceanic ecosystems implemented by a jurisdiction to prevent and minimize the introduction, establishment, and spread of NIMS, thereby protecting the economy, environment, and human well-being from NIMS damaging impacts in its jurisdictional regions (Olenin et al. 2011, Carvalho et al. 2023, Bland et al. 2024). The importance of this global issue is clearly illustrated by the number of international conventions and guidelines that have been established at various levels to address the challenges that NIMS pose (e.g., see table S1).

**Figure B1.**
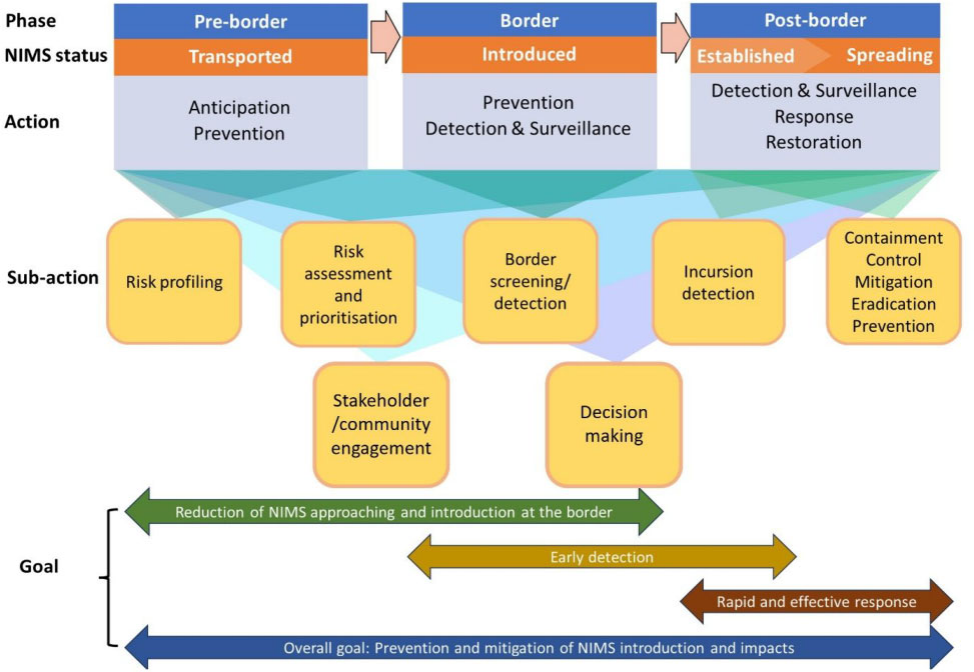
Marine biosecurity continuum. “Phase” represents both management and marine bioinvasion stages. “Action” represents management activities corresponding to each phase. “Sub-action” is the subcategory of management actions and can be implemented at different management stages. “Goal” refers to the target outcomes of management activities. Note: The list of sub-actions is not exhaustive.

To effectively manage introduced species, strategies need to be established and implemented. Typical marine biosecurity strategies are along a continuum (figure B1). That includes three stages, namely pre-border (where NIMS are in transport), border (NIMS are being introduced), and post-border (NIMS are established and spread), with management activities aiming for different outcomes (broad goals) (Bland et al. 2024). Border in this context refers to the boundary of a jurisdiction.

Our search in Web of Science (WoS) and Scopus databases using multiple combinations of search terms related to AI in marine biosecurity, including “AI” or “artificial intelligence,” “machine learning,” “deep learning,” “neural network,” “automation,” or stemming words, in conjunction with “marine biosecurity” or “marine invasive species” with “invasive” being replaced by other common terminologies used in this field (e.g., non-native, nonnative, non-indigenous, exotic, pest, or alien) or “hull fouling,” “biofouling” in the marine/coastal context. Although we employed a broad and carefully considered range of search terms across multiple databases and platforms covering their entire publication span (from database inception through to our search date), we acknowledge that some relevant publications may have been missed. Nonetheless, we consider our search to be comprehensive in scope with respect to the terms and parameters applied.

The search was conducted on the 24th of February 2025 on the topic in the databases (i.e., Title, Abstract, and Keywords). We included only research articles focused on AI development and applications that address marine biosecurity issues presented in figure B1. Review articles and papers that are not focused on AI in marine biosecurity were deliberately excluded from our review scope. The search terms were then used to search Google Scholar on the 25th of February 2025. The articles shown in the first ten Google Scholar pages were evaluated based on the same inclusion and exclusion criteria applied to the two scientific databases. The detailed search strategies and PRISMA flowchart (figure S1) are presented in Supplemental Information S1. A total of 25 relevant papers were included in the final synthesis, as presented in table S2 of the Supplemental Material. Although the publications are extensive through time, the first paper identified using our search terms was in 2005.

In the following sections, we analyze key findings from the literature review, outlining existing efforts and gaps in AI-driven marine biosecurity research. We also explore the available resources and emerging opportunities for leveraging AI, from real-time threat detection to automated ecosystem monitoring. However, the implementation of AI in this domain faces ethical and practical hurdles, including concerns over data privacy, algorithmic biases, and the complexity of policy adaptation, which we discuss. The outcome of this analysis is a structured roadmap for AI advancement, emphasizing interdisciplinary collaboration, regulatory frameworks, and scalable technology solutions. Our conclusion ties these findings and recommendations together to reaffirm the critical role that AI can play in safeguarding marine environments while addressing the challenges that must be overcome for its responsible deployment.

## AI applications in marine biosecurity

**Figure 1.**
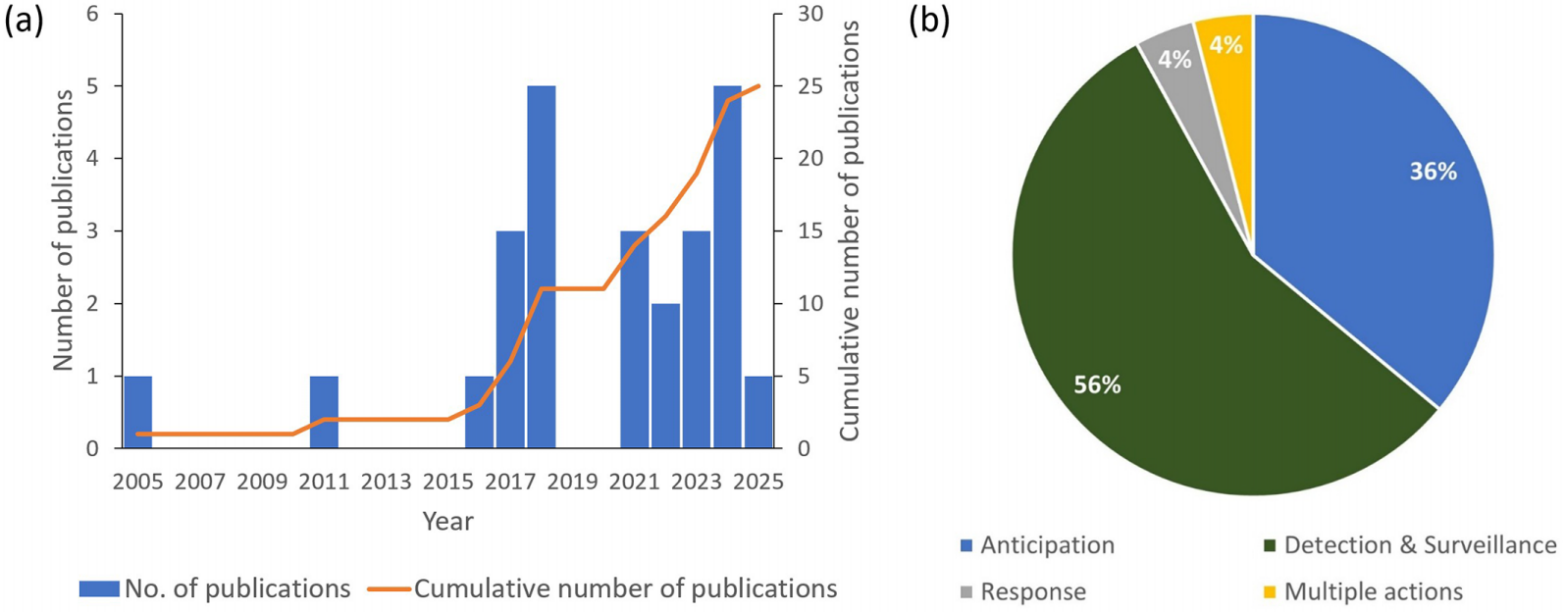
Overview of the reviewed literature on AI applications in marine biosecurity: (a) time series of annual publication counts (bars) and cumulative publications (line), and (b) proportional distribution of the reviewed studies across four marine biosecurity management actions.

Despite the growing applications of AI in the marine field (Cruz et al. 2021, Song et al. 2023, Gesami and Nunoo 2024), AI-enabled approaches have not been widely applied to the management of NIMS. Since the work of Floerl and colleagues (2005), research on this topic has been published sporadically, showing short upward trends during 2016–2018 and 2022–2024, but no publication during 2006–2010, 2012–2016, and 2019–2020 (figure 1a).

NIMS-related AI-based approaches can be grouped into four categories, including Anticipation, Detection and Surveillance, Response Decisions, and Multiple Actions, spanning across the three stages of invasions and management (i.e., pre-border, border, and post-border; figure B1). Most AI applications in the marine biosecurity literature fall into the Detection and Surveillance category (14 papers), followed by Anticipation (9 papers), while Response Decisions and Multiple Actions were each addressed by one study (figure 1b).

### Anticipation

Within the Anticipation category, the reviewed research was categorized into two groups, including risk profiling (four papers), and risk assessment and prioritization (five papers) (table S3). Risk was identified and profiled in terms of high-volume shipping routes (Shi et al. 2024, Song et al. 2024), invasion spread power (Shi et al. 2024), the risk factors of inspection failures (Clarke et al. 2017), and levels of hull fouling (Floerl et al. 2005). Risk profiling publications focused on the shipping pathway, with vessels being the primary vector, examining multiple ports at national (Floerl et al. 2005, Clarke et al. 2017), or global scales (Shi et al. 2024, Song et al. 2024).

AI-based risk assessments focused on known NIMS by predicting their survival (Holland et al. 2021), dispersal (Pontin et al. 2011), and habitat suitability (i.e., ecological niches) (Miller 2016, Coro et al. 2018, López-Reyes et al. 2023) in novel environments in contemporary and future climate conditions. The number of target species examined in these publications ranged from an individual species (Pontin et al. 2011, Miller 2016, Coro et al. 2018) to multiple species, with 26 species in López-Reyes and colleagues (2023) and 49 in Holland and colleagues (2021).

Although these studies illustrate the capability of AI techniques to support targeted monitoring and resource allocation, their predictive performance remains constrained by the challenges of capturing the complex drivers of marine bioinvasion risks (see table S2).

### Detection and Surveillance

Most studies on AI-powered identification and detection of NIMS were based on image data (12 out of 14 papers) (table S4). Data used for each study were sourced from multiple data platforms, with the size of training datasets ranging from 100 to 438,657 images. Image data types were diverse, including video-derived, static, and synthetic images. Acoustic data (Demertzis et al. 2018) and e-DNA (Demertzis and Iliadis 2017) were each used in a single study to detect non-native species. This demonstrates the potential of AI using diverse data types and sources to enhance early detection capacity.

Biofouling (i.e., the attachment and accumulation of marine species to any parts of submerged structures such as vessels, marine debris, aquaculture farms, offshore oil rigs, or renewable energy devices) is one of the primary pathways/vectors of NIMS (Lewis and Coutts 2009, Hewitt and Campbell 2010, Campbell et al. 2017, Chan et al. 2022). Identification and detection of biofouling accounts for 50% of the papers in the Detection and Surveillance grouping. Biofouling detection mainly sought to identify common macrofouling organisms such as barnacles, mussels, algae, oysters, and tunicates (Chin et al. 2017, Gormley et al. 2018, O’Byrne et al. 2018, Santos et al. 2022, Signor et al. 2023). The other studies focused on detecting the presence of biofouling (classified as “Fouled” or “Clean”) (Rashid et al. 2024) or the severity (scale/level/amount) of biofouling (Mannix et al. 2021) on submerged structures. Effective detection of fouling organisms and severity levels can inform timely management interventions, reducing the likelihood of species establishment.

The main targets of AI-powered detection studies were “notorious” invasive NIMS, with most of the analyzed papers focusing on an individual species. Prime examples are sun coral (*Tubastraea sp*.; Luz et al. 2025), lionfish (*Pterois volitans*; Martínez-González et al. 2021), tubeworm/fanworm (*Sabella spallanzanii*; Tait et al. 2023), and the invasive macroalgae (*Rugulopteryx okamurae*; Roca et al. 2022). Multiple invasive species were targeted in two studies (Demertzis et al. 2018, Mifsud Scicluna et al. 2024).

Although some authors developed their machine learning models for biofouling or NIMS detection, others used existing frameworks such as YOLO (You Only Look Once), CoralNet, and ResNet for real-time species detection, automated image annotation, and deep feature extraction, respectively (table S4). The availability of existing tools creates opportunities to accelerate deployment and reduce development costs, making AI-based detection and monitoring more accessible for regulatory authorities and industry stakeholders.

### Response decision support

Only one identified publication in our search addressed an AI-based approach to support response decision-making. Xiao and colleagues (2018) applied machine learning to predict the likelihood of success in eradicating aquatic invasive species. The data input was sourced from the peer-reviewed literature, with features including taxonomy, invasion status, habitat type, area occupied, eradication method, and containment measures. Although model accuracy was moderate (65%–78% across the five machine learning models; Xiao et al. 2018), the study demonstrates how AI can guide managers in prioritizing eradication efforts and allocating resources where success is most likely.

### AI-supported NIMS management system (multiple actions)

Katsanevakis and colleagues (2024) present the only multidisciplinary initiative to date that systematically explores the use of AI in managing invasive non-native species (INNS), including NIMS, across all stages of invasion and management (pre-border, border, and post-border). At the pre-border stage, they propose employing a large language model (LLM) to enhance data search and integration on invasive species, thereby improving risk profiling. For border control, an AI-powered smartphone application could assist officers in recognizing and intercepting INNS. Both border and post-border biosecurity would further benefit from AI-enabled automated surveillance that integrates diverse data types, including imagery, acoustics, and eDNA.

Risk assessments throughout the biosecurity continuum are envisioned to be strengthened by an AI-based workflow, incorporating a native-invasive interaction database to identify vulnerable and threatened species and predictive distribution models to forecast future hotspots of INNS impacts. Although these applications remain at the proposal stage, the study provides a framework for how AI could be embedded across the biosecurity continuum, outlining underlying principles and assessing readiness levels (Katsanevakis et al. 2024).

## Opportunities for AI development and implementation in marine biosecurity

Although current AI-enabled methods have demonstrated applicability in marine biosecurity, there remains substantial scope for further development and implementation. Key opportunities include refining existing models and expanding target variables across three levels of biosecurity (species, vectors, and pathways). Improved identification and understanding of NIMS species traits, vectors, and pathways are essential for risk profiling, resource prioritization, and early detection, thereby supporting more effective management strategies (e.g., Campbell 2009, Hewitt and Campbell 2010, Williams et al. 2013, Azmi et al. 2014, Keith et al. 2016, Tidbury et al. 2016, Laeseke et al. 2020, Bayley et al. 2024).

Advances in AI algorithms, coupled with the increasing availability of marine datasets, provide strong foundations for scalable solutions. Marine science has long generated extensive data, with recent exponential growth in both volume and complexity driven by sensor and genomic technologies (Malde et al. 2020). Ongoing digitalization and the expansion of public data portals further enhance accessibility, creating favorable conditions for the integration of AI into marine biosecurity.

### Anticipation

The increasing availability and accessibility of NIMS data are reflected in ongoing efforts to integrate multi-source records into compiled platforms, enabling streamlined access through online tools and interoperable web services. Notable examples include the World Register of Introduced Marine Species (WRiMS; Costello et al. 2021), European Alien Species Information Network (EASIN; Katsanevakis et al. 2015), and the Global Invasive Species Database (GISD; Invasive Species Specialist Group ISSG 2015). Consequently, NIMS records have become increasingly detailed. For example, GISD species profiles now include habitat descriptions, ecological traits, pathways of introduction and spread, biological status, impacts on biodiversity and ecosystem services, and management options (e.g., see www.iucngisd.org/gisd/howto.php). Such data are critical for species-level risk profiling and assessment while also providing insights into their potential pathways and vectors of introduction.

Data on key pathways and vectors have similarly expanded. The automatic identification system (AIS), widely used in maritime navigation, now provides real-time vessel movement data (Lee et al. 2019, Yang et al. 2019), with AIS-based deep learning models tested for forecasting, imputation, anomaly detection, and classification in the marine industry (Li et al. 2024). High-quality AIS data are available commercially through sources such as Lloyd’s List Intelligence (www.lloydslistintelligence.com/about-us/data-and-analytics/ais-seaorbis). Emerging vectors, including marine debris, are also increasingly documented (“DeNIS”–Canning-Clode et al. 2023). These datasets form a foundation for marine biosecurity.

Risk analysis models could be improved by incorporating vessel types and associated biosecurity risks (Bereza et al. 2023), organism entrainment rates, species life history traits, historical pathways, invasion timing, and taxonomic group-specific data (Wonham and Carlton 2005, Hilliam et al. 2024). Predictive models could further integrate real-time shipping data and environmental variables influencing NIMS survival and establishment, such as source-destination similarity (Tzeng et al. 2024). Collectively, these advances in data availability provide a strong basis for developing more robust and accurate AI models in marine biosecurity.

### Detection and surveillance

Current AI-powered detection methods for NIMS remain limited, often focussing on a small number of target NIMS, typically charismatic or visible species (see table S4). Reducing this taxonomic bias requires expanding detection across broader groups and integrating diverse data types. For visual data, extensive labeled image repositories are now available, including Wildfish++ (103,034 images of 2348 fish species; Zhuang et al. 2020), FishNet (94,532 images of 17,357 aquatic species; Khan et al. 2023), FathomNet (100,000 + images; Katija et al. 2022), iNaturalist (1000s of species with expert-verified labels; www.inaturalist.org), FishBase (www.fishbase.org), and SeaLifeBase (www.sealifebase.org). Coupled with advanced object detection, such as YOLO (You Only Look Once; Redmon et al. 2016), DETR (Detection Transformers; Carion et al. 2020), and AI-powered applications like SuperFish (Pudaruth et al. 2021), Fishify (Dhore et al. 2024), WikiFish (Elbatsh et al. 2021), iBivalves (Maravillas et al. 2022), CoralNet (Beijbom et al. 2012), and others (Zhou et al. 2025), these resources provide a strong foundation for AI-based NIMS detection. Where annotated datasets are lacking, photorealistic synthetic imagery offers a promising alternative (O’Byrne et al. 2018).

Beyond visual approaches, AI models can be trained on auditory data (Demertzis et al. 2018), leveraging marine acoustics as a complementary detection tool (Risch and Parks 2017, Mooney et al. 2020). Acoustic monitoring can overcome barriers such as depth, turbidity, and low light and is particularly effective for cryptic species (Lammers et al. 2008, McCauley et al. 2017). Large acoustic databases, including those of NOAA Passive Acoustic Data archived by the National Center for Environmental Information (NCEI; www.ncei.noaa.gov/products/passive-acoustic-data), the International Quiet Ocean Experiment (https://iqoe.org/), Monterey Bay Aquarium Research Institute (MBARI) Open Acoustic Data (www.mbari.org/project/open-acoustic-data/), the National Marine Mammal Foundations Cetacean Hearing Database (https://nmmf.org/our-work/biologic-bioacoustic-research/cetacean-hearing-database/), and Global Library of Underwater Biological Sounds (GLUBS; www.glubs.org/), provide valuable resources for training detection models that can be tagged to underwater acoustic network (e.g., the United Kingdom Acoustics Network).

For NIMS that are cryptic, present in low densities, or phenotypically similar to native species, genomic approaches now offer a highly sensitive, cost-effective set of tools that allow for much earlier detection than was previously possible (Sherman et al. 2016, Bowers et al. 2021, Flitcroft et al. 2025). In particular, environmental DNA approaches (eDNA—genetic materials that organisms leave behind in the environment) represent several advantages (Rees et al. 2014, Ardura 2019, Wasik and Pattinson 2024).

Species-specific qPCR assays and broader community metabarcoding workflows are now routinely deployed for marine biosecurity surveillance and are generating large amounts of data (e.g., Zaiko et al. 2018, Wiltshire et al. 2023). To fully realize the value of these genomics approaches, however, key limitations still need to be addressed, including improved DNA reference libraries, standardized best practice guidelines, clearer metadata standards, and much greater automation (De Brauwer et al. 2023, Rishan et al. 2023, Sepulveda et al. 2023, Jarman et al. 2024). Critically, AI can help solve many of these bottlenecks, meaning that the integration of genomic and eDNA datasets with AI represents one of the largest untapped opportunities for accelerating and improving marine biosecurity decision-making and species detection (Rishan et al. 2023, Jarman et al. 2024, Gupta et al. 2026). Specifically, AI can help interpret complex eDNA and genomic datasets for more reliable and rapid species identifications, reduce uncertainty at low signal detection, accelerate new assay development, and couple real-time genomic surveillance with shipping data to model risk (Rishan et al. 2023, Jarman et al. 2024, Gupta et al. 2026).

Although targeted qPCR and metabarcoding workflows have increasingly applied algorithmic or probabilistic inference to genetic outputs (e.g., McClenaghan et al. 2020, Rishan et al. 2023, Nichols et al. 2025), the deliberate and standardized integration of AI with genetic datasets for NIMS detection remains limited, with documented applications, to date, confined to a single-species case study (Demertzis and Iliadis 2017). However, the approach is scalable to detect multiple species simultaneously (Zhang et al. 2023). DNA barcode databases, such as Barcode of Life Data System (BOLD; Ratnasingham and Hebert 2007), GenBank (Sayers et al. 2019), National Biodiversity DNA Library (NBDL; https://nbdl.csiro.au/), and complementary continental or national repositories are essential for this work. Autonomous eDNA sampling platforms have also been developed, significantly expanding spatial and temporal monitoring capacity (Van Wyngaarden et al. 2024). For robust AI applications, it is important that genetic datasets are standardized, quality-controlled, and machine-readable, retaining quantitative outputs, replicate structure, and explicit uncertainty (Ficetola et al. 2015, Libbrecht and Noble 2015, Whalen et al. 2022).

Detection performance could be further enhanced by using multi-modal models incorporating multiple forms of information, such as text (e.g., the literature, metadata), bioacoustics, and images (Sahraoui et al. 2023). Additionally, detected species may not always be limited to known NIMS. To determine whether a species is native or non-native, its occurrence location can be compared with its known natural range (Demertzis et al. 2018). This approach is particularly important in monitoring programs, as it helps identify previously unrecorded species that may pose a biosecurity risk. eDNA is already being coupled with biophysical models to help resolve local movement, dispersal shadows, and detection limits (Ellis et al. 2022). However, progress is still constrained by the spatial and temporal resolution of available hydrodynamic models. AI is likely to rapidly improve both the scale and resolution of these models, enabling more accurate identification of eDNA sources and movement pathways. This, in turn, will support the development of surveillance strategies that maximize detection probability, and more targeted follow-up surveys when detections of NIMS occur.

Beyond supervised learning, where model training requires large, annotated datasets, alternative AI methods can offer promise. For example, semi- or weakly supervised learning (as applied in Demertzis and Iliadis 2017) reduces the need for extensive labeling, unsupervised learning can uncover patterns without labels (Ghahramani 2004), and reinforcement learning adapts through interaction and feedback (Sutton and Barto 1998). Although these methods may not identify species directly, they are valuable for detecting anomalies in marine environments, especially when annotated data are limited or too costly to obtain.

### Decision-making support and public engagement

At all biosecurity stages, decision-making should be largely informed using AI-powered decision support tools. Shafiq and colleagues (2024) demonstrate how decision support systems powered by AI can improve invasive species management through their capacity to analyze vast amounts of data, make data-based recommendations, and facilitate adaptive management. In marine biosecurity, where decisions are often made with incomplete data (Campbell et al. 2018), AI-enabled risk profiling, risk assessment, and early detection ensure that high-risk species, pathways, and entry points (e.g., ports) are identified, and resources are efficiently allocated by prioritizing high-risk threats. At a post-border stage, the success of response options could be predicted using machine learning models (e.g., Xiao et al. 2018). This approach would aid managers in weighing management options (e.g., eradication, control, containment) when an incursion occurs.

Public and stakeholder engagement is also vital across the marine biosecurity continuum. Engaging the public aids in early detection (Hourston et al. 2015) and enhances social legitimacy and acceptability of management decisions (McKinley and Fletcher 2011, Uffman-Kirsch et al. 2020, Campbell et al. 2024). Effective public engagement should be two-way as a deliberative process (Revill and Jefferson 2013, McAllister et al. 2020). To that end, natural language processing offers an innovative way to enhance social learning (Goñi et al. 2023), and narrative building (i.e., a process that uses deliberation to create shared understandings of issues) to facilitate public participation and engagement (Marmolejo-Ramos et al. 2022). Moreover, although still in an early stage, LLMs have been developed to explain AI-generated recommendations, improving human users’ understanding of underlying model outcomes (Dwivedi et al. 2023, Said 2025).

## Challenges

Although AI holds great promise for marine biosecurity, it is not without its challenges. One of the critical challenges lies in data availability, accessibility, quality, and standardization (Stahl 2021). Collecting data from marine environments is inherently difficult, and while automated devices can help, they may be a vector of NIMS, if not properly managed (Thaler et al. 2015). Despite progress in marine data that includes NIMS, datasets remain fragmented, varied in type and quality, and stored in various repositories (Malde et al. 2020). The lack of standardized, AI-ready training datasets represents a challenge that is intended to be addressed by AI itself (Sagi et al. 2020, Ditria et al. 2022). Moreover, marine biosecurity-relevant data are unevenly distributed worldwide, with limited information from regions like the Southern Hemisphere (Costello et al. 2021, Nuñez et al. 2022, Carvalho et al. 2023). Incomplete, unbalanced, biased, or poor-quality data can impair AI model performance and reduce the generalizability of AI predictions (Balahur et al. 2022).

Another challenge concerns the high computational and financial demands of AI model development and deployment (e.g., Cottier et al. 2024, Luccioni et al. 2024). Many factors influence AI development costs, including AI model complexity, data requirements (quantity, variety, quality), data collection, cleaning, annotation, and storage, development team expertise, integration, and deployment (Lukianchenko and Teres 2024). This represents a prohibitive obstacle for AI development and deployment in countries with limited resources, raising critical equity concerns in global AI development. Improving equity, generalization, and scalability in AI solutions requires addressing the structural gaps in data availability, computational infrastructure, and human capital that disproportionately affect the “Global South” (He 2025). Achieving equitable participation, therefore, depends not only on local investment but also on targeted external support for capacity-building, infrastructure development, and enhanced representation in AI research and governance (Thiébaut 2024).

Although AI has been utilized to address environmental sustainability (Nti et al. 2022), its development and deployment come with significant environmental costs. These costs are primarily due to energy and fresh water required to train and operate large-scale AI systems, and consequent emissions (e.g., Dauvergne 2022, Crawford 2024). This paradox calls for pragmatic strategies to reduce AI’s ecological footprint.

Ethical concerns related to data privacy, accountability, fairness, and potential technology misuse also arise. Training AI model for marine biosecurity needs vast amounts of data, such as eDNA data (Flitcroft et al. 2025, Gupta et al. 2026), vessel movements (Bereza et al. 2023, Shi et al. 2024), port management (Hewitt et al. 2011), proprietary and personal data for risk profiling (Mannix et al. 2024), and public information for communication and engagement strategies (Amin et al. 2025). These data can be sensitive and require careful handling. These concerns often entail social resistance, as algorithmic decision-making may be perceived as illegitimate, especially for far-reaching decisions (Duberry and Duberry 2022). In Australia, for example, public trust in AI remains low (Lockey et al. 2020), and people are sceptical of the government’s ability to responsibly manage personal data (Biddle et al. 2018). The characteristics of AI, including complexity (i.e., complex algorithms and big data), opacity (resulting from the complexity, and partly from deliberate choice/policy), and interdependence (i.e., liability and responsibility for the final decision become diffused when it is based on numerous datasets from different sources), raise further questions on the legitimacy of algorithmic decision-making (Lepri et al. 2018, Grimmelikhuijsen and Meijer 2022). Additionally, incomplete or biased datasets can inadvertently disadvantage certain communities, sectors, or regions (e.g., Borines et al. 2025, Bose 2025, Mergen et al. 2025, Ziegler et al. 2025). The important questions become whether the benefits and burdens of marine biosecurity are distributed fairly, and whether algorithmic decisions are justifiable in the eyes of ocean-dependent industries and communities, and the public, especially when these decisions affect livelihoods or access to marine resources. Ethical AI development and deployment, therefore, demands careful attention to data privacy protection, fairness, and transparency with equitable access to information.

The integration of AI into marine biosecurity systems also raises political concerns about authority, governance, and the shifting balance of power over information, surveillance, and regulatory decisions, especially when those decisions entail high-stakes regulatory and economic consequences. The distinction between “governance of AI” (i.e., governance structures to regulate and steer AI development and deployment) and “governance by AI” (i.e., AI adopted as part of governance mechanisms) creates a justificatory hierarchy of authority (Erman and Furendal 2024). The possession of data and resources determines the power of actors in the AI era (Drakopulos et al. 2022), leading to an asymmetry of power between a small group of states and corporations that have control over advanced technologies, and the majority who do not (Korkmaz 2024, Akıllı 2025). Assumptions and choices are embedded in AI designs, which are prone to manipulation (Vassilev et al. 2024, Basol 2025). Examples of choices in marine biosecurity may include which species count as “high risk,” which ports or industries are more prioritized, which risk threshold justifies restrictions on industrial operations or trade, and whose values and uncertainties matter. Those kinds of choices are subject to conflicting interests, visions, and values among different stakeholders (e.g., Campbell et al. 2016, Gollan and Barclay 2021, Le and Campbell 2022). Any errors, such as species misidentification, detection bias, geographical bias, or malevolent misuse, may risk compromising economic stability and sparking geopolitical tensions at national, regional, or global scales.

These concerns can interplay and hinder effective AI adoption in marine biosecurity.

## A roadmap for AI implementation

**Figure 2.**
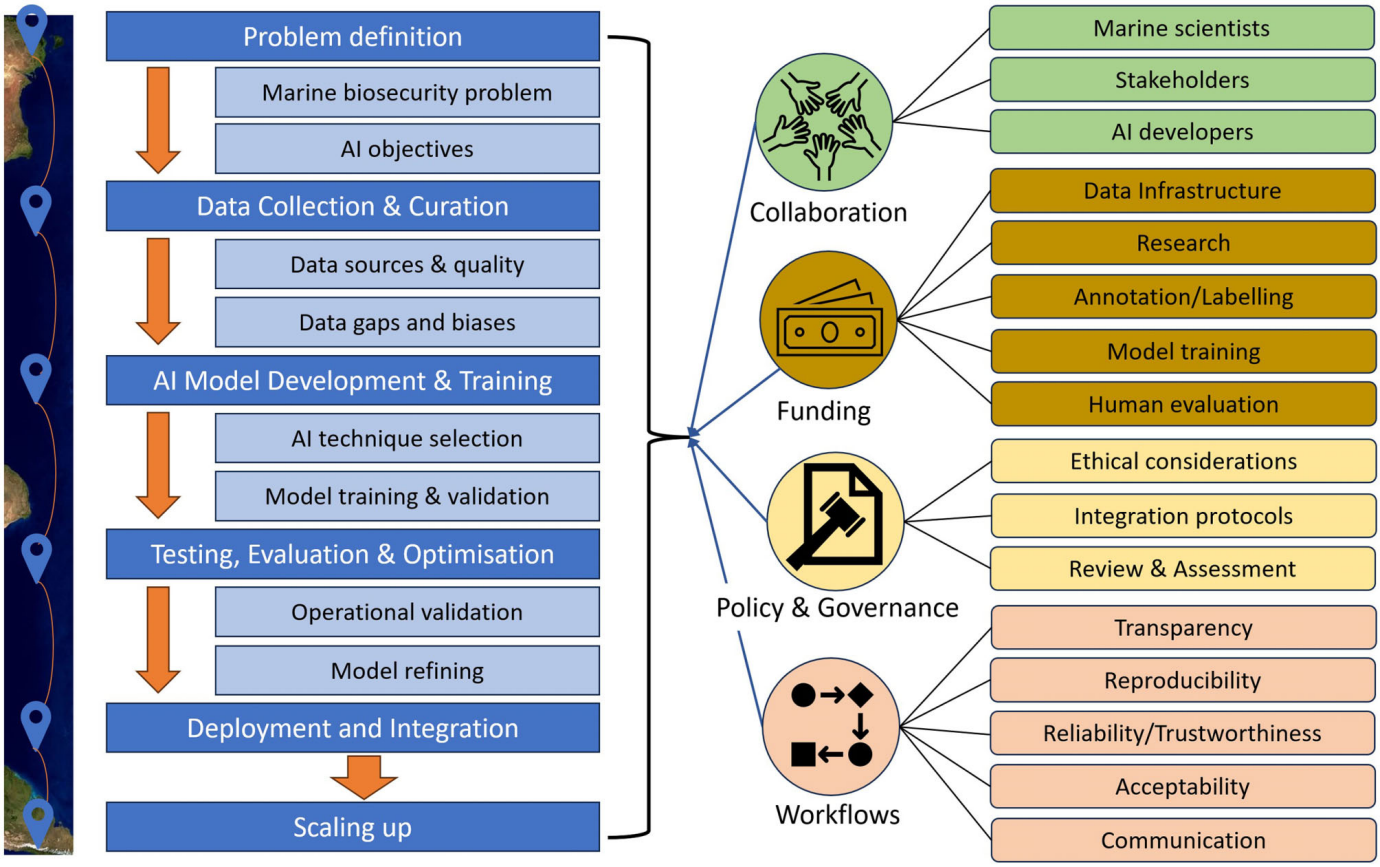
Multi-stage approach to AI implementation in marine biosecurity.

Realizing the potential of AI in marine biosecurity, while navigating its challenges, calls for a structured approach grounded in four enabling pillars: collaboration, funding, policy, and workflows (depicted in figure 2).

The first step involves defining the specific marine biosecurity task that AI is intended to address (e.g., risk identification/profiling, early detection of NIMS, impact prediction, response option selection). Although the task may initially be identified by marine biosecurity managers, collaboration with key stakeholders is essential in determining whether AI is an appropriate and feasible solution (Sturm et al. 2021). Beyond marine scientists and AI developers, whose expertise is essential for designing AI solutions in marine biosecurity, key stakeholders include marine-dependent industries and local populations, such as fishers, aquaculture operators, coastal communities, and Indigenous communities. Early engagement with these stakeholders not only benefits from locally grounded knowledge but also ensures that the proposed solutions are relevant and legitimate, facilitating smoother adoption and implementation when technologies are deployed (McDonald et al. 2020, Record et al. 2022, Hounshell et al. 2025).

The availability of data and computational resources will guide the choice of the AI techniques and necessitate the development of a data-for-AI framework (e.g., Fagnan et al. 2019, Esteva et al. 2020, Rothenberger et al. 2020, Kotta et al. 2024). This involves data design (i.e., identifying and documenting data sources), data sculpting (ensuring data quality through selection, cleaning, and annotation), and evaluation data (data for AI model validation) (Liang et al. 2022). Collaboration between AI developers, marine scientists, and other stakeholders throughout this pipeline helps mitigate bias and enhance scalability and robustness.

Once trained and validated, the AI model should undergo operational validation in real-world settings. At this stage, expert input, particularly from marine research scientists, is critical for human evaluation of model predictions, ensuring the reliability of the AI solution before integration into marine biosecurity protocols. International collaboration and cooperation are vital for scaling AI solutions (i.e., deploying more computational power, using larger datasets, and building bigger models) and ensuring generalizability across regions or systems.

Throughout AI solution development, Funding, Policy & Governance, and Workflows are critical enablers. Given the high financial and computational demands, sustained investment is needed across key areas, including data pipeline, technical infrastructure, model training, interdisciplinary research, and expert annotation and validation. Importantly, AI development must be guided by strong governance and policy frameworks, ensuring adherence to ethical standards, incorporating principles of diversity, equity, and inclusion (Abrams 2024, The United Nations 2025), mitigation of environmental impacts (van Wynsberghe 2021, Crawford 2024), transparent AI implementation and integration protocols (e.g., protocols for use, user training, data management strategies), and ongoing review mechanisms (Stahl 2021).

A growing suite of AI ethics guidelines, frameworks, and standards has emerged across scales and sectors (see table S5, and Jobin et al. 2019), providing guidance for the ethical and responsible development and deployment of AI in marine biosecurity. Global ethical principles for AI emphasize human rights and democratic values, including justice, fairness, transparency, explainability, and the robustness and reliability of AI systems. Alongside this, responsible data governance is guided by the FAIR principles (Findability, Accessibility, Interoperability, and Reusability) for data in general, and the CARE principles (Collective Benefit, Authority to Control, Responsibility, and Ethics) for the governance of Indigenous data (table S5). To foster trust and adoption of AI solutions, the workflow must be well-documented and communicated. Transparency, reproducibility, reliability, and communicability are key to gaining stakeholder and policymaker acceptance and support (Davis et al. 2024).

## Conclusion

Although AI has been recognized as a powerful tool in the marine field, AI applications in marine biosecurity remain underdeveloped, with a limited number of early stage, or project-based implementations, and no documented evidence of their operational deployment. There are opportunities for AI development and integration into marine biosecurity risk identification, early NIMS detection, predictive risk assessment, and informed decision-making. However, unlocking the potential of AI in marine biosecurity (and other contexts) requires recognizing and overcoming critical challenges, including data limitation, high financial and computational costs, ethical and social concerns around data privacy, and responsible use of AI technology.

We note that the AI-enabled approaches described in this paper are not comprehensive in scope. Marine biosecurity is a multi-dimensional domain that spans ecological, economic, social, and regulatory dimensions (Briski et al. 2013), and its complexity opens various opportunities for AI to address. Moreover, as AI technologies are evolving quickly, the landscape of available tools and their potential applications is rapidly shifting. The roadmap that we propose in this paper recommends continued collaboration, investments, responsibility, and transparency for trustworthy and sustainable AI development and implementation.

## Supplementary Material

biag049_Supplemental_Files

## Notes

Marnie L. Campbell (marnie.campbell@ecu.edu.au) is the Executive Dean of the School of Science at Edith Cowan University. She is a leading expert in marine biosecurity and ecosystem restoration, with extensive experience in risk assessment and management strategies. Chi T. U. Le (thaiuyenchi.le@ecu.edu.au) is a Postdoctoral research fellow at Edith Cowan University, Australia. She specializes in statistical modeling, machine learning, and stakeholder analysis, with applications in marine invasive species and socio-environmental systems. Jumana Abu-Khalaf (j.abukhalaf@ecu.edu.au) is a researcher and senior lecturer in Computer Science at Edith Cowan University, specializing in robotics and artificial intelligence. Her work spans multiple disciplines and recently expanded to applying AI techniques to address marine environmental challenges. Craig Sherman (craig.sherman@deakin.edu.au) is the Associate Head of the School of Life and Environmental Sciences (Research and Industry Engagement) at Deakin University. His expertise spans ecology, genetics, zoology, evolutionary biology, and climate change impacts and adaptation, with a focus on the marine environment.
