# Supplementary material for: Leveraging artificial intelligence to enhance marine biosecurity
Source: Bioscience. 2026 May 1;76(8):690–703. doi: 10.1093/biosci/biag049 (PMC13486004; doi:10.1093/biosci/biag049)
Supplement: Supplementary file 1 [file biag049_supplemental_files.zip › Supplemental Material.docx]

**Supplemental Material**

**Title:** **Leveraging Artificial Intelligence to Enhance Marine Biosecurity**

**S1. Search strategy and PRISMA flow chart for the literature review**

**Web of Science search**

TS=(((AI OR "artificial intelligence" OR "machine learning" OR "deep learning" OR automa* OR autonomous OR “neural network*”) AND ("marine biosecurity" OR “invasive marine species" OR "non-native marine species" OR "nonnative marine species" OR "marine bioinvasion*" OR "alien marine species" OR “non-indigenous marine species" OR “introduced marine species” OR “nonindigenous marine species” OR "marine invasive species" OR "marine non-native species" OR "marine nonnative species" OR "marine bioinvasion*" OR "marine alien species" OR “marine non-indigenous species" OR “marine introduced species” OR “marine nonindigenous species” OR “marine pest*” OR (marine AND (biofouling OR "hull fouling")))))

Date: 24/02/2025 – Results = 113

**Scopus Search**

Article title, Abstract, Keywords = (((AI OR "artificial intelligence" OR "machine learning" OR "deep learning" OR automa* OR autonomous OR “neural network*”) AND ("marine biosecurity" OR “invasive marine species" OR "non-native marine species" OR "nonnative marine species" OR "marine bioinvasion*" OR “marine invasion*” OR "alien marine species" OR “non-indigenous marine species" OR “introduced marine species” OR “nonindigenous marine species” OR "marine invasive species" OR "marine non-native species" OR "marine nonnative species" OR "marine bioinvasion*" OR "marine alien species" OR “marine non-indigenous species" OR “marine introduced species” OR “marine nonindigenous species” OR “marine pest*” OR “marine biofouling” OR "hull fouling"))))

Date: 24/02/2025

Results = 143

**After removing duplications**: 155

**Inclusion criteria:**

- Being peer-reviewed research article where AI methods are described and discussed
- Supporting marine biosecurity decision-making in the actions/sub-actions along its continuum

**Exclusion criteria:**

- Being literature review or recommendation in nature
- Related to platform technologies such as autonomous underwater vehicles (AUVs) and remotely operated vehicles (ROVs)

**Figure S1:** Flow diagram for the literature review (adapted to Page et al., 2021)

**Table S1**: International conventions and guidelines at various levels that address non-native (marine) species

| **Title** | **Scale** | **Type** | **Relevance** | **Reference Link** |
| --- | --- | --- | --- | --- |
| Convention on Biological Diversity (CBD) | International | Convention | Encourages prevention, control, or eradication of invasive species threatening biodiversity. | <https://www.cbd.int/> |
| Ballast Water Management (BWM) Convention | International | Convention | Aims to prevent spread of harmful aquatic organisms via ships' ballast water. | <https://www.imo.org/en/About/Conventions/Pages/International-Convention-for-the-Control-and-Management-of-Ships'-Ballast-Water-and-Sediments-(BWM).aspx> |
| IMO Biofouling Guidelines | International | Guideline | Guidance on minimizing transfer of invasive species via vessel hull biofouling. | <https://www.imo.org/en/OurWork/Environment/Pages/Biofouling.aspx> |
| FAO Code of Conduct for Responsible Fisheries | International | Guideline | Promotes responsible introductions and management of aquatic species. | <https://www.fao.org/fisheries/code/en> |
| Global Invasive Species Programme (GISP) | International | Programme | Provides global frameworks and tools for managing invasive species. | <http://www.gisp.org/> |
| SPREP Invasive Species Guidelines | Regional (Pacific) | Guideline | Supports Pacific Island nations in managing invasive species, including marine pests. | <https://www.sprep.org/invasive-species-management> |
| COBSEA Action Plan on Marine and Coastal Invasive Species | Regional (East Asia) | Action Plan | Promotes regional cooperation to combat marine invasives. | <https://www.unep.org/cobsea> |
| HELCOM Baltic Sea Action Plan | Regional (Baltic Sea) | Action Plan | Includes commitments to manage and prevent invasive species introductions. | <https://helcom.fi/about-us/convention/> |
| OSPAR Convention for the NE Atlantic | Regional (NE Atlantic) | Convention | Addresses marine biodiversity threats including invasive species. | <https://www.ospar.org/> |
| EU Regulation on Invasive Alien Species (1143/2014) | Regional (EU) | Regulation | Sets rules for prevention and management of invasive species across EU member states. | <https://ec.europa.eu/environment/nature/invasivealien/index_en.htm> |
| Barcelona Convention | Regional (Mediterranean) | Convention | Recognises the threat of invasive species to biodiversity, addresses key pathways for species introduction, and promotes cooperation, monitoring, and prevention. | <https://www.unep.org/unepmap/who-we-are/barcelona-convention-and-protocols> |
| Australia's Biosecurity Act 2015 | National (Australia) | Legislation | Framework for managing biosecurity threats including marine invasive species. | <https://www.legislation.gov.au/Details/C2015A00061> |
| National System for Marine Pest Incursions | National (Australia) | Management System | National coordination of marine pest prevention and response. | <https://www.marinepests.gov.au/> |
| Australian Ballast Water Management Requirements | National (Australia) | Regulation | Implements BWM Convention to prevent marine pest spread. | <https://www.amsa.gov.au/marine-environment/ballast-water> |
| Australian Biofouling Management Guidelines | National (Australia) | Guideline | Voluntary guidelines to manage vessel biofouling. | <https://www.marinepests.gov.au/marine_pests/biofouling_guidelines> |
| Victoria’s Invasive Plants and Animals Policy Framework | State (Victoria, Australia) | Management Plan | State-level policy framework for managing invasive species, including marine pest threats. | <https://agriculture.vic.gov.au/biosecurity/protecting-victoria/legislation-policy-and-permits/invasive-plants-and-animals-policy-framework> |
| NSW Marine Estate Management Strategy | State (NSW, Australia) | Strategy | Includes marine biodiversity protection and invasive species control. | <https://www.marine.nsw.gov.au/strategy> |
| WA Biosecurity and Agriculture Management Act 2007 | State (WA, Australia) | Legislation | Regulatory framework for biosecurity including marine invasives. | <https://www.legislation.wa.gov.au/legislation/statutes.nsf/main_mrtitle_10236_homepage.html> |
| China's Biosecurity Law 2021 | National (China) | Legislation | Prohibits unauthorised introductions of alien species and mandates strengthened monitoring and treatment efforts. | <http://english.mee.gov.cn/News_service/media_news/202106/t20210616_839242.shtml> |
| China's Wildlife Protection Law (Amended 2022) | National (China) | Legislation | Emphasises the prevention of invasive species | <https://www.fao.org/faolex/results/details/en/c/LEX-FAOC173552/> |
| China's Regulations on the Quarantine of Imported and Exported Animals and Plants (1982) | National (China) | Regulation | Focuses on the quarantine of foreign animals and plants entering the country | <https://www.fao.org/faolex/results/details/en/c/LEX-FAOC213164/> |
| China's Marine Environmental Protection Law | National (China) | Legislation | Prevents the introduction of invasive alien marine species | <https://doi.org/10.1007/978-3-031-56218-1_16> |
| Canada’s Aquatic Invasive Species Regulations | National (Canada) | Regulation | Provides legal framework to prevent and manage aquatic invasive species. | <https://laws-lois.justice.gc.ca/eng/regulations/SOR-2015-121/> |
| Canada's Shipping Act 2001 | National (Canada) | Legislation | Regulates shipping activities, including measures to prevent the introduction and spread of non-native aquatic species | <https://laws-lois.justice.gc.ca/eng/acts/c-10.15/> |
| New Zealand Biosecurity Act 1993 | National (New Zealand) | Legislation | Comprehensive legislation for exclusion and management of invasive species. | <https://www.legislation.govt.nz/act/public/1993/0095/latest/DLM314623.html> |
| US National Invasive Species Act (NISA) 1996 | National (USA) | Legislation | Regulates ballast water to control aquatic invasive species. | <https://www.congress.gov/bill/104th-congress/house-bill/3217> |
| US Lacey Act | National (USA) | Legislation | Addresses illegal wildlife trade and prevent the introduction of harmful non-native species | <https://www.fws.gov/law/lacey-act> |
| UK's Wildlife and Countryside Act 1981 | National (UK) | Legislation | Addresses the introduction and management of non-native species | <https://www.legislation.gov.uk/ukpga/1981/69/enacted> |
| UK's The Invasive Alien Species (Enforcement and Permitting) Order 2019 | National (UK) | Legislation | establishes offences, enforcement provisions, and permitting requirements related to invasive alien species in England, Wales, and the UK's offshore marine area | <https://www.legislation.gov.uk/uksi/2019/527/contents> |

**Table S2:** (provided in a separate file)

**Table S3: AI applications in marine biosecurity - Anticipation**

| Sub-action | AI methods | Model evaluation | Papers |
| --- | --- | --- | --- |
| Risk profiling | Rule-based algorithm | Not specified | Shi et al. (2024) |
|  | Deep learning models | 84.8% Accuracy | Song et al. (2024) |
|  | Machine learning | 22.1% explained variation, 43% misclassification  Trade-off between Sensitivity and Specificity | Floerl et al. (2005)  Clarke et al. (2017) |
| Risk assessment and prioritisation | - Artificial Neural Network – Multi-layer perceptron (MLP) - Versatile Quantum-inspired Evolutionary Algorithm (vQEA) | >80% Accuracy | Pontin et al. (2011) |
|  | Unsupervised neural networks – Self-organising maps | Not specified | Miller (2016) |
|  | - Artificial neural networks (ANN) - Maximum Entropy (Maxent) - Support Vector Machine (SVM) | 79% - 96% Accuracy | Coro et al. (2018) |
|  | Gradient boosted machine learning (XGBoost) | >90% Accuracy | Holland et al. (2021) |
|  | - Maxent - Minimum volume ellipsoid (MVE) - Generalised linear model (GLM) | Not specified | López-Reyes et al. (2023) |

**Table S4: AI applications used in marine biosecurity – Detection and Surveillance**

| Target species | AI methods | Input data | Size of training dataset | Model Evaluation | Papers |
| --- | --- | --- | --- | --- | --- |
| Biofouling | CNN | Static images | 1825 | >70% accuracy | Chin et al. (2017) |
|  | CoralNet | Video footage | 857 | 33% - 37% accuracy | Gormley et al. (2018) |
|  | SegNet + SVM | Synthetic images | 2,500 | 94% accuracy | O'Byrne et al. (2018) |
|  | CNN | Static images | 10,263 | 89% agreement | Mannix et al. (2021) |
|  | WNN | RGB images | 438,657 | 95% accuracy | Santos et al. (2022) |
|  | CNN | Static images | 1261 | 69% accuracy | Signor et al. (2023) |
|  | YOLO version 8 | Video footage | 1800 | 97.3% accuracy | Rashid et al. (2024) |
| Sun Coral (*Tubastraea* sp.) | YOLO version 8 | Static images | 500 | >90% accuracy | Luz et al. (2025) |
| Tubeworm (*Sabella spallanzanii*) | YOLO version 3 | Static images | 100 | ~ 70% detection efficacy | Tait et al. (2023) |
| *Rugulopteryx okamurae* | SVM | Remote sensing images | N/A | >91% accuracy | Roca et al. (2022) |
| Lionfish *Pterois volitans* | YOLO version 4  SSD-Mobilenet-v2 | Static images | 2818 | 63.66% recall, 89.79% precision, and 79.15%  mAP | Martínez-González et al. (2021) |
| Five common invasive fish species | CNN  ResNet18  YOLO version 8 | Static images | 1337 | 57%, 91% and 84%, accuracy respectively | Mifsud Scicluna et al. (2024) |
| Silver toadfish *Lagocephalus sceleratus* | Semi-supervised learning | eDNA | 856 | 91.3% accuracy | Demertzis and Iliadis (2017) |
| Ten fish species + eight mammal species | Deep extreme learning machine | Acoustics | 3073 sounds | >87% accuracy | Demertzis et al. (2018) |

*Note:* CNN: Convolutional neural network; SVM: Support vector machine; WNN: Wide neural network.

**Table S5**: AI Ethics guidelines

| **Document name** | **Year** | **Scale/Level** | | **Category** | **Key contents** | **Reference link** |
| --- | --- | --- | --- | --- | --- | --- |
| UNESCO Recommendation on the Ethics of AI | 2021 | Global (UN) | Principles & Policy baseline | | Global standard adopted by 194 Member States, promoting fundamental principles such as transparency and fairness, and highlighting the importance of human oversight of AI systems. | <https://www.unesco.org/en/articles/recommendation-ethics-artificial-intelligence> |
| Council of Europe Framework Convention on AI | 2024 | Regional (Europe, open globally) | Legal governance, rights protection | | The Framework Convention covers the use of AI systems by public authorities, including private actors acting on their behalf, and private actors. The activities within the lifecycle of AI system must comply with seven fundamental principles, including:  1) Human dignity and individual autonomy 2) Equality and non-discrimination 3) Respect for privacy and personal data protection 4) Transparency and oversight 5) Accountability and responsibility 6) Reliability 7) Safe innovation | <https://www.coe.int/en/web/artificial-intelligence/the-framework-convention-on-artificial-intelligence> |
| OECD AI Principles | 2019 | Intergovernmental (OECD/G20) | Policy baseline and evaluation metrics | | First intergovernmental standard on AI, consisting of five value-based principles and five recommendations that provide practical and flexible guidance for policy-makers and AI actors. Values-based principles include: Human rights and democratic values, including fairness and privacy; Transparency and explainability; Robustness, security, and safety; and Accountability. | <https://www.oecd.org/en/topics/ai-principles.html> |
| EU Artificial Intelligence Act (Regulation 2024/1689) | 2024 | Regional (EU) | Regulatory and compliance framework | | Risk classification, conformity assessment, enforcement | <https://eur-lex.europa.eu/eli/reg/2024/1689/oj/eng> |
| UK ICO Guidance on AI & Data Protection | 2023 | National (UK) | Privacy and data ethics framework | | Data protection, fairness, explainability, risk mitigation | <https://ico.org.uk/for-organisations/uk-gdpr-guidance-and-resources/artificial-intelligence/guidance-on-ai-and-data-protection/> |
| Singapore Model AI Governance Framework | 2020 | National (Singapore) | Operational governance guidance | | Accountability, testing, human oversight, transparency | [www.pdpc.gov.sg/-/media/Files/PDPC/PDF-Files/Resource-for-Organisation/AI/SGModelAIGovFramework2.pdf](http://www.pdpc.gov.sg/-/media/Files/PDPC/PDF-Files/Resource-for-Organisation/AI/SGModelAIGovFramework2.pdf) |
| Canada Directive on Automated Decision-Making | 2021 | National (Canada) | Public-sector algorithmic accountability | | Impact assessment, documentation, transparency in government use of AI | <https://publications.gc.ca/collections/collection_2021/sct-tbs/BT48-31-2021-eng.pdf> |
| ISO/IEC 23894:2023 (AI Risk Management) | 2023 | International (ISO/IEC) | Risk management process | | Hazard identification, evaluation, and mitigation across AI lifecycle | <https://www.iso.org/standard/77304.html> |
| ISO/IEC 42001:2023 (AI Management System) | 2023 | International (ISO/IEC) | AI governance management system | | Policy, leadership, and continual improvement framework for AI systems | <https://www.iso.org/standard/42001> |
| NIST AI Risk Management Framework (AI RMF 1.0) | 2023 | National (USA) | Voluntary operational risk framework | | Mapping, measuring, managing, and governing AI risks | <https://www.nist.gov/itl/ai-risk-management-framework> |
| IEEE Ethically Aligned Design (EAD) | 2019 | International (IEEE) | Ethical design principles | | Human rights, transparency, accountability, and well-being | <https://standards.ieee.org/wp-content/uploads/import/documents/other/ead1e_general_principles.pdf> |
| WHO Guidance on Ethics & Governance of AI for Health | 2021 | Sectoral (Health, Global) | Health sector ethics & governance | | Fairness, safety, accountability, inclusiveness in health AI | <https://www.who.int/publications/i/item/9789240029200> |
| ITU/WHO AI for Health Initiative (FG-AI4H / GI-AI4H) | 2018 | Sectoral (Health, Global) | Benchmarking and standards development | | Evaluation, standardization, and governance of health AI models | <https://aiforgood.itu.int/reports_publications/shaping-ethics-regulation-and-standardization-in-ai-for-health-itu-who-focus-group-on-ai-for-health/> |
| UNICEF Policy Guidance on AI for Children | 2020 | Sectoral (Children, Global) | Child-centered ethical AI guidance | | Equity, safety, privacy, and child rights in AI | <https://www.unicef.org/innocenti/reports/policy-guidance-ai-children> |
| FAIR Data Principles | 2016 | Cross-sector (Global) | Data stewardship & transparency principles | | Findability, accessibility, interoperability, and reusability of data | <https://www.go-fair.org/fair-principles/> |
| CARE Principles for Indigenous Data Governance | 2019 | Cross-sector (Global, Indigenous data) | Equitable and ethical data governance | | Collective benefit, authority to control, responsibility, and ethics | <https://www.gida-global.org/care> |
| African Union Data Policy Framework | 2022 | Regional (Africa) | Data governance and sovereignty policy | | Data sovereignty, interoperability, and cross-border governance | <https://au.int/en/documents/20220728/au-data-policy-framework> |

**Reference**

Chin, C. S., Si, J. T., Clare, A. S., & Ma, M. D. (2017). Intelligent Image Recognition System for Marine Fouling Using Softmax Transfer Learning and Deep Convolutional Neural Networks [Article]. *Complexity*, 9, Article 5730419. 10.1155/2017/5730419

Clarke, S., Hollings, T., Liu, N., Hood, G., & Robinson, A. (2017). Biosecurity risk factors presented by international vessels: a statistical analysis. *Biological Invasions*, *19*(10), 2837-2850. 10.1007/s10530-017-1486-1

Coro, G., Vilas, L. G., Magliozzi, C., Ellenbroek, A., Scarponi, P., & Pagano, P. (2018). Forecasting the ongoing invasion of Lagocephalus sceleratus in the Mediterranean Sea. *Ecological Modelling*, *371*, 37-49. <https://doi.org/10.1016/j.ecolmodel.2018.01.007>

Demertzis, K., & Iliadis, L. (2017). Detecting invasive species with a bio-inspired semi-supervised neurocomputing approach: the case of Lagocephalus sceleratus. *Neural Computing and Applications*, *28*(6), 1225-1234. <https://doi.org/10.1007/s00521-016-2591-2>

Demertzis, K., Iliadis, L. S., & Anezakis, V.-D. (2018). Extreme deep learning in biosecurity: the case of machine hearing for marine species identification. *Journal of Information and Telecommunication*, *2*(4), 492-510. <https://doi.org/10.1080/24751839.2018.1501542>

Floerl, O., Inglis, G. J., & Hayden, B. J. (2005). A Risk-Based Predictive Tool to Prevent Accidental Introductions of Nonindigenous Marine Species. *Environmental management*, *35*(6), 765-778. 10.1007/s00267-004-0193-8

Gormley, K., McLellan, F., McCabe, C., Hinton, C., Ferris, J., Kline, D. I., & Scott, B. E. (2018). Automated image analysis of offshore infrastructure marine biofouling [Article]. *Journal of Marine Science and Engineering*, *6*(1), Article 2. 10.3390/jmse6010002

Holland, O., Shaw, J., Stark, J. S., & Wilson, K. A. (2021). Hull fouling marine invasive species pose a very low, but plausible, risk of introduction to East Antarctica in climate change scenarios [Article]. *Diversity and Distributions*, *27*(6), 973-988. 10.1111/ddi.13246

López-Reyes, K., Osorio-Olvera, L., Rojas-Soto, O., Chiappa-Carrara, X., Patrón-Rivero, C., & Yáñez-Arenas, C. (2023). An exhaustive evaluation of modeling ecological niches above species level to predict marine biological invasions [Article]. *Marine Environmental Research*, *186*, 9, Article 105926. 10.1016/j.marenvres.2023.105926

Luz, A. C. N., Barroso, V. R., Batista, D., Lessa, A. A., Coutinho, R., & Xavier, F. C. (2025). Early detection of marine bioinvasion by sun corals using YOLOv8. *Intelligent Marine Technology and Systems*, *3*(1), 2. 10.1007/s44295-024-00052-1

Mannix, E., Wei, S., A. Woodham, B., Wilkinson, P., & Robinson, A. P. (2021). Automating the assessment of biofouling in images using expert agreement as a gold standard. *Scientific Reports*, *11*(1), 2739. <https://doi.org/10.1038/s41598-021-81011-2>

Martínez-González, Á. T., Ramírez-Rivera, V. M., Caballero-Vázquez, J. A., & Jáuregui, D. A. G. (2021). Deep learning algorithm as a strategy for detection an invasive species in uncontrolled environment. *Reviews in Fish Biology and Fisheries*, *31*(4), 909-922. <https://doi.org/10.1007/s11160-021-09667-7>

Mifsud Scicluna, B., Gauci, A., & Deidun, A. (2024). AquaVision: AI-Powered Marine Species Identification. *Information*, *15*(8), 437. 10.3390/info15080437

Miller, K. B. (2016). Forecasting at the edge of the niche: *Didemnum vexillum* in Southeast Alaska [Article]. *Marine Biology*, *163*(2), 12, Article 30. 10.1007/s00227-015-2799-1

O'Byrne, M., Pakrashi, V., Schoefs, F., & Ghosh, B. (2018). Semantic segmentation of underwater imagery using deep networks trained on synthetic imagery [Article]. *Journal of Marine Science and Engineering*, *6*(3), Article 93. 10.3390/jmse6030093

Page, M. J., McKenzie, J. E., Bossuyt, P. M., Boutron, I., Hoffmann, T. C., Mulrow, C. D., Shamseer, L., Tetzlaff, J. M., Akl, E. A., Brennan, S. E., Chou, R., Glanville, J., Grimshaw, J. M., Hróbjartsson, A., Lalu, M. M., Li, T., Loder, E. W., Mayo-Wilson, E., McDonald, S., . . . Moher, D. (2021). The PRISMA 2020 statement: an updated guideline for reporting systematic reviews. *BMJ*, *372*, n71. 10.1136/bmj.n71

Pontin, D. R., Schliebs, S., Worner, S. P., & Watts, M. J. (2011). Determining factors that influence the dispersal of a pelagic species: A comparison between artificial neural networks and evolutionary algorithms [Article]. *Ecological Modelling*, *222*(10), 1657-1665. 10.1016/j.ecolmodel.2011.03.002

Rashid, H., Habbouche, H., Amirat, Y., Mamoune, A., Titah-Benbouzid, H., & Benbouzid, M. (2024). B-FLOWS: Biofouling Focused Learning and Observation for Wide-Area Surveillance in Tidal Stream Turbines [Article]. *Journal of Marine Science and Engineering*, *12*(10), Article 1828. 10.3390/jmse12101828

Roca, M., Dunbar, M. B., Román, A., Caballero, I., Zoffoli, M. L., Gernez, P., & Navarro, G. (2022). Monitoring the marine invasive alien species Rugulopteryx okamurae using unmanned aerial vehicles and satellites [Original Research]. *Frontiers in Marine Science*, *9*. 10.3389/fmars.2022.1004012

Santos, J., Pedersen, M. L., Ulusoy, B., Weinell, C. E., Pedersen, H. C., Petersen, P. M., Dam-Johansen, K., & Pedersen, C. (2022). A Tunable Hyperspectral Imager for Detection and Quantification of Marine Biofouling on Coated Surfaces [Article]. *Sensors*, *22*(18), Article 7074. 10.3390/s22187074

Shi, H., Wang, C., Zhao, H., Wang, S., & Chen, Y. (2024). Bioinvasion risk analysis based on automatic identification system and marine ecoregion data. *High-Confidence Computing*, *4*(4), 100210. <https://doi.org/10.1016/j.hcc.2024.100210>

Signor, J., Schoefs, F., Quillien, N., & Damblans, G. (2023). Automatic classification of biofouling images from offshore renewable energy structures using deep learning [Article]. *Ocean Engineering*, *288*, Article 115928. 10.1016/j.oceaneng.2023.115928

Song, R., Spadon, G., Pelot, R., Matwin, S., & Soares, A. (2024). Enhancing global maritime traffic network forecasting with gravity-inspired deep learning models. *Scientific Reports*, *14*(1), 16665. 10.1038/s41598-024-67552-2

Tait, L. W., Bulleid, J., Rodgers, L. P., Seaward, K., Olsen, L., Woods, C., Lane, H., & Inglis, G. J. (2023). Towards remote surveillance of marine pests: A comparison between remote operated vehicles and diver surveys [Article]. *Frontiers in Marine Science*, *10*, 13, Article 1102506. 10.3389/fmars.2023.1102506
