# Supplementary material for: Leveraging artificial intelligence to enhance marine biosecurity
Source: Bioscience. 2026 May 1;76(8):690–703. doi: 10.1093/biosci/biag049 (PMC13486004; doi:10.1093/biosci/biag049)
Supplement: Supplementary file 1 [file biag049_supplemental_files.zip › Table S2- Summary of the reviewed papers.docx]

| **Author** | **Year** | **Title** | **Objectives** | **Marine biosecurity action** | **Management sub-action** | **Management stage** | **Species** | **Number of species** | **Location** | **AI methods** | **Dataset** | **Data source** | **Applications** | **Limitations** | **Future Research** |
| --- | --- | --- | --- | --- | --- | --- | --- | --- | --- | --- | --- | --- | --- | --- | --- |
| Pontin et al. | 2011 | Determining factors that influence the dispersal of a pelagic species: A comparision between artificial neutral networks and evolutionary algorithms | Predicting the dispersal of passive marine species (*Physalia spp.*) using oceanographic data and machine learning models. | Anticipation | Risk assessment and prioritisation | Pre-border, Post-border | Jellyfish *Physalia sp*. (Phylum Cnidaria and class Siphonophora). | 1 | New Zealand | - The study compares two AI methods for feature selection: a multilayer perceptron (MLP), which is a type of artificial neural network, and the Versatile Quantum-inspired Evolutionary Algorithm (vQEA), an evolutionary algorithm designed to handle complex and noisy ecological data sets. | - The study utilized oceanographic data on wave height and wind direction.  - Records of occurrences of Physalia on swimming beaches during the summer season were included. | - The oceanographic data used in the study was sourced from the National Oceanic and Atmospheric Administration/National Centers for Environmental Prediction (NOAA/NCEP) Wavewatch III model hindcast output, which represented eighty 1.25 × 1 degree global grid cells surrounding New Zealand. - Oceanographic data on wave height and wind direction, along with records of occurrences of Physalia on swimming beaches throughout the summer season were included. | The study identifies two independent systems influencing the distribution of Physalia around New Zealand, driven by oceanographic variables. This enhances understanding of passive dispersers. | - The lack of surface current information limits the ability to generalize the importance of selected features in the models. - Daily observation data necessitated averaging, preventing exploration of diurnal patterns and short-duration phenomena. - Inadequate sampling may misrepresent species presence or absence, leading to class imbalance in datasets. - The quality and quantity of data significantly impact model effectiveness, as noted in the paper. | - Future research could explore the generalization of models beyond the limited areas previously studied for Physalia occurrence.  - Investigating the ecological impacts of oceanographic conditions on Physalia presence in various regions may provide valuable insights.  - Further studies could assess the effectiveness of different feature selection methods in ecological modeling.  - Research could focus on improving data quality and sampling methods to enhance model accuracy in predicting species distributions.  - Exploring the integration of molecular data with ecological models could yield a deeper understanding of species distribution patterns. |
| Miller | 2016 | Forecasting at the edge of the niche: Didemnum vexillum in Southeast Alaska | - Assessing potential habitats for the invasive species *Didemnum vexillum* in Southeast Alaska using temperature and salinity factors. - Creating maps predicting the potential spread of *D. vexillum* for invasion risk assessment and management actions. | Anticipation | Risk assessment and prioritisation | Pre-border | *Didemnum vexillum* | 1 | Southeast Alaska | - The study employed a self-organizing map (SOM) - an unsupervised neural network that visualizes clusters in multidimensional data while preserving the similarity between samples. | - The study utilized the Tongass National Forest streams dataset for stream mouth locations.  - ShoreZone intertidal habitat data was used for habitat classification.  - Average surface temperature and salinity data were sourced from various locations, including Whiting Harbor, AK.  - The Alaska ShoreZone dataset provided geomorphic characteristics of the intertidal coastline.  - The study also referenced data from the USGS Woods Hole Coastal and Marine Science Center.  - Environmental conditions in Whiting Harbor were evaluated to create temperature and salinity scenarios. | - The datasets used in the study were sourced from various locations. The worldwide locations of Didemnum vexillum infestation were obtained from the United States Geological Survey (USGS) Woods Hole Coastal and Marine Science Center website.  - Additionally, average conductivity and temperature data were collected from profiles by the International Pacific Halibut Commission in Southeast Alaska for the years 2010 and 2011.  - Measured summer temperature and salinity data for Southeast Alaska were compiled from 617 stations sampled between 1998 and 2011.  - Furthermore, winter temperature data were compiled from thermographs from 16 bays in Southeast Alaska and from NOAA tide and current stations for December-February.  - The study also utilized data from nearshore seine surveys conducted between 1998 and 2009. | - The paper discusses using self-organizing maps to assess potential habitat for Didemnum vexillum in Southeast Alaska.  - It highlights the value of maps predicting invasive species spread for assessing invasion risk and management actions.  - The research emphasizes the need for mechanistic models incorporating life history data for accurate habitat assessment.  - It addresses the challenges of risk assessment modeling when species move to new environments.  - The study suggests that existing distribution may not represent the full range of conditions for species survival. | - The existing distribution of *D. vexillum* may not represent its full fundamental niche, limiting understanding of its habitat preferences.  - There is uncertainty in using current distribution to predict potential spread in new environments at the species' tolerance edge. | - Future research could focus on the low-temperature tolerance limits of *D. vexillum* over extended periods.  - Investigating *D. vexillum*'s salinity tolerance in various environments is necessary.  - More studies on the phenotypic plasticity of invasive tunicates could provide insights.  - Understanding the full range of conditions affecting *D. vexillum*'s fitness is essential.  - Research on the anthropogenic factors influencing *D. vexillum*'s spread is warranted. |
| Coro et al. | 2018 | Forecasting the ongoing invasion of Lagocephalus sceleratus in the Mediterranean Sea | Forecasting the invasion (i.e., the future geographical distribution) of the silver-cheeked toadfish, *Lagocephalus sceleratus*, in the Mediterranean Sea. | Anticipation | Risk assessment and prioritisation | Pre-border, Post-border | Cheeked toadfish (*Lagocephalus sceleratus*) | 1 | Mediterranean Sea | - The study utilized Artificial Neural Networks (ANNs) as the primary AI method | - The study utilized 263 Mediterranean occurrence records, combining SPD records and literature reviews from 2003 onwards.  - Environmental parameters included depth, distance from land, and water temperature, constrained to 18 features.  - The AquaMaps Consortium provided eco-geographical parameters likely associated with species habitat preferences.  - The datasets were used to evaluate model performance rather than for training. | - The datasets used in the study are sourced from environmental data attached to the presence and pseudo-absence locations of the invasive species Lagocephalus sceleratus. This data was processed at a resolution of 0.5°, which is the minimal resolution for the models employed in the research. - The environmental information was associated with these locations through the DataMiner's "occurrence-enrichment" process, which produced a CSV file containing an enriched dataset. | The method is applicable to monitor cheektoad fish and other invasive species in various regions. | - ANN is hard to intuitively understand.  - MaxEnt is sensitive to data bias, affecting its performance with unreliable presence records.   - The dynamic model's performance is lower compared to the geographical reachability distribution, indicating potential limitations in prediction accuracy.  - Overestimation of presence locations may lead to misleading conclusions. | - Future research should focus on the ecological and economic impacts of L. sceleratus in the Mediterranean Sea.  - Strategies for selective fishing to manage pufferfish populations require further investigation.  - The application of ecological niche models (ENMs) to predict invasive species distribution needs more exploration.  - Understanding the biotic and abiotic factors influencing L. sceleratus habitats is essential for future studies.  - The potential spread of L. sceleratus into the Black Sea warrants additional research.  - The integration of cloud computing-based algorithms in ecological studies presents opportunities for further methodological advancements. |
| Holland et al. | 2021 | Hull fouling marine invasive species pose a very low, but plausible, risk of introduction to East Antarctica in climate change scenarios | Predicting species survival in shallow benthic ecosystems in current conditions, and under future climate scenarios. | Anticipation | Risk assessment and prioritisation | Border, Post-border | Global list of known NIMS | 49 | Australian East Antarctica and subantarctic islands | - The study employs gradient boosted machine learning (XGBoost), to predict the survival of known hull fouling marine invasive species in shallow benthic ecosystems adjacent to Australia's Antarctic research stations and subantarctic islands. - The model utilizes four important environmental variables: sea surface temperature, salinity, nitrate, and pH, to develop predictions regarding suitable environments for potentially invasive species. |  | - The datasets used in the study were sourced from the Ocean Biogeographic Information System (OBIS), which provided location data for marine invasive species (MIS). | - The study predicts potential hull fouling marine invasive species in East Antarctica and subantarctic islands using machine learning models. - It identifies species that could survive in shallow benthic ecosystems adjacent to Australian Antarctic research stations. - The research emphasizes the need for surveillance and monitoring to prevent invasive species establishment. - It discusses the importance of understanding environmental suitability and factors influencing species establishment. | - The study is based on an incomplete record of global species presence, potentially missing species at higher latitudes.  - Predictions may represent errors in the model or indicate borderline environmental suitability for the species.  - The ability of species to reach new locations and reproduce successfully is considered unlikely based on current knowledge.  - The assumption that sea surface temperature is a key barrier to marine invasive species may be flawed.  - The study highlights the need for further investigation into environmental variables and their synergistic effects on species survival. | - Further research is needed on fouling community composition in hull and protected niche areas to understand marine invasions better.  - The study suggests a precautionary approach for surveillance and monitoring in East Antarctica due to potential risks from hull fouling species.  - Investigating the propagule load carried by research and resupply vessels is essential to assess invasion risks.  - Understanding the reproductive success of species in these environments could provide insights into their potential invasiveness. |
| Lopez-Reyes et al. | 2023 | An exhaustive evaluation of modeling ecological niches above species level to predict marine biological invasions | Evaluating the effectiveness of supraspecific modeling units for predicting invasive species distributions in marine ecosystems. | Anticipation | Risk assessment and prioritisation | Pre-border, Post border | N/A | 26 | Global | - The study utilized three different AI methods for ecological niche modeling: the maximum entropy algorithm (Maxent), the minimum volume ellipsoid (MVE), and the generalized linear model (GLM). | - 12 pre-processed global raster surfaces from Bio-Oracle 2.0, with a cell resolution of approximately 10 km².  - Environmental data included abiotic factors such as temperature and salinity. - The presence records of 26 target species, grouped with phylogenetically related species.  - Global raster data of oceanic currents were incorporated to refine accessibility polygons. | - The datasets used in the study were sourced from Bio-Oracle 2.0, which provided 12 pre-processed global raster surfaces with a cell resolution of approximately 10 km². These surfaces describe relevant environmental variables for marine organisms, specifically focusing on temperature and salinity data from 2000 to 2014. | - Ecological niche modeling applications in climate change and biological invasions, enhancing understanding of species' threats under environmental changes.  - The construction of supraspecific modeling units is evaluated for improving predictions of invasive species distributions.  - Correlative niche modeling could be used as a tool for estimating species distributions. | - The algorithm may make commission errors when predicting species responses under novel environmental conditions.  - The models only considered abiotic factors, potentially overlooking important biotic interactions.  - Species not in environmental equilibrium lead to assumptions about their responses, complicating predictions.  - The predictive ability of correlative models depends on the extent of the species' potential niche.  - The presence of closely related species may skew model estimates towards their occupied niches. | N/A |
